# Biomechanical study of internal fixation methods for femoral neck fractures based on Pauwels angle

**DOI:** 10.3389/fbioe.2023.1143575

**Published:** 2023-03-03

**Authors:** Fuyang Wang, Yuchen Liu, Yi Huo, Ziming Wang, Jinge Zhang, Minghao Xu, Kaiming Ma, Linbao Wang, Yongtao Lu, Liangliang Cheng, Dewei Zhao

**Affiliations:** ^1^ Department of Orthopedics, Affiliated Zhongshan Hospital of Dalian University, Dalian, China; ^2^ Department of Engineering Mechanics, Dalian University of Technology, Dalian, China; ^3^ Department of anesthesiology, Affiliated Zhongshan Hospital of Dalian University, Dalian, China

**Keywords:** femoral neck fractures, finite element analysis, biomechanics, medial buttress plate, cannulated compression screws, porous tantalum screws

## Abstract

**Objective:** To select the most appropriate internal fixation method based on the Pauwels angle, in order to provide a new concept for clinical accurate treatment of femoral neck fractures (FNFs).

**Methods:** FNFs models of Pauwels 30° ; 40° ; 50° ; 60° were created respectively. For Pauwels ≤ 50° , 1, 2 and 3 Cannulated Compression Screws (CCS) and Porous Tantalum Screws (PTS) were used to fix the fracture for the models. For Pauwels 60° , 3CCS and Medial Buttress Plate (MBP) combined with 1, 2 and 3CCS were used to fix the fracture. Based on the results of the finite element (FE) analysis, the biomechanical properties of each model were compared by analyzing and evaluating the following four parameters: maximal stress of the bone (MBS), maximal stress of the implants (MIS), maximal displacement of bone (MBD), interfragmentary motion (IFM).

**Results:** At Pauwels 30° , the larger parameters were found in 1CCS, which was 94.8 MPa (MBS), 307.7 MPa (MIS), 0.86 mm (MBD) and 0.36 mm (IFM). In 2CCS group, the parameters were 86.1 MPa (MBS), 254.4 MPa (MIS), 0.73 mm (MBD) and 0.27 mm (IFM), which were similar to those of PTS. At Pauwels 40° ; 50° , with the increase of the number of used CCS, accordingly, the parameters decreased. Particularly, the MIS (Pauwels 50° ) of 1CCS was 1,195.3 MPa, but the other were less than the yield range of the materials. At Pauwels 60° , the MBS of 3CCS group was 128.6 Mpa, which had the risk of failure. In 2CCS + MBP group, the parameters were 124.2 MPa (MBS), 602.5 MPa (MIS), 0.75 mm (MBD) and 0.48 mm (IFM), The model stability was significantly enhanced after adding MBP.

**Conclusion:** Pauwels type Ⅰ (< 30° ) fractures can reduce the number of CCS, and PTS is an appropriate alternative treatment. For Pauwels type Ⅱ fractures ( 30°∼50° ), the 3CCS fixation method is still recommended. For Pauwels type Ⅲ fractures (> 50° ), it is recommended to add MBP to the medial femoral neck and combine with 2CCS to establish a satisfactory fracture healing environment.

## 1 Introduction

**FIGURE 1:**
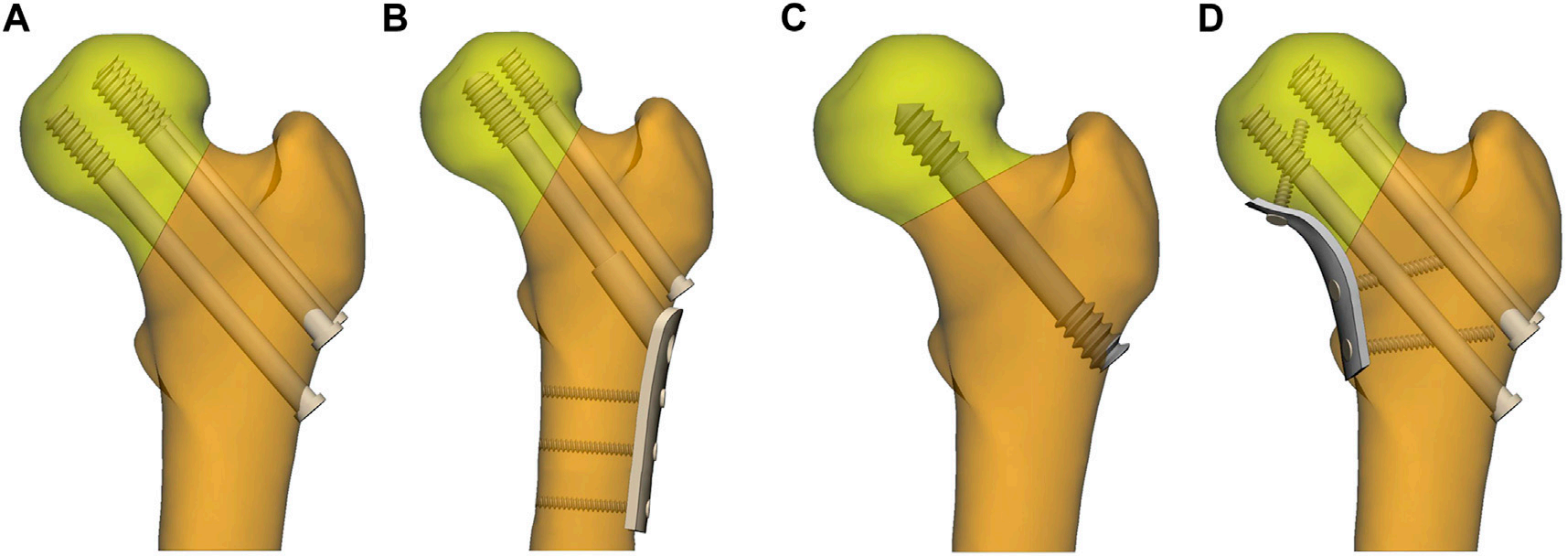
Internal fixation of femoral neck fractures. **(A)** three parallel cannulated compression screws (CCS); **(B)** dynamic hip screws and anti-rotation screws; **(C)** porous tantalum screws (PTS); **(D)** three parallel cannulated compression screws and medial buttress plate (MBP).

FNFs account for 3.6% of the total fractures and 53% of hip fractures (Thorngren et al., 2002). Due to its special anatomical structure and biomechanical complexity, the selection of internal fixation should be as small as possible and have little interference with the blood supply, and more importantly, it should provide sufficient stability. At present, the mainstream internal fixation methods in the clinical applications include: cannulated compression screws (CCS) and dynamic hip screw (DHS) (Figures 1A,B) (Li et al., 2018). However, the current conventional fixation method interferes greatly with the blood supply in the femoral head (Zhao et al., 2017), and shows insufficient shear force for Pauwels type Ⅲ fracture (Li et al., 2019b). The rate of bone non-union after internal fixation is as high as 10%–34%, and the rate of osteonecrosis is as high as 35%–48%. Therefore, it has always been a difficult problem in orthopedic treatment (Davidovitch et al., 2010; Nauth et al., 2017; Medda et al., 2019).

**FIGURE 2:**
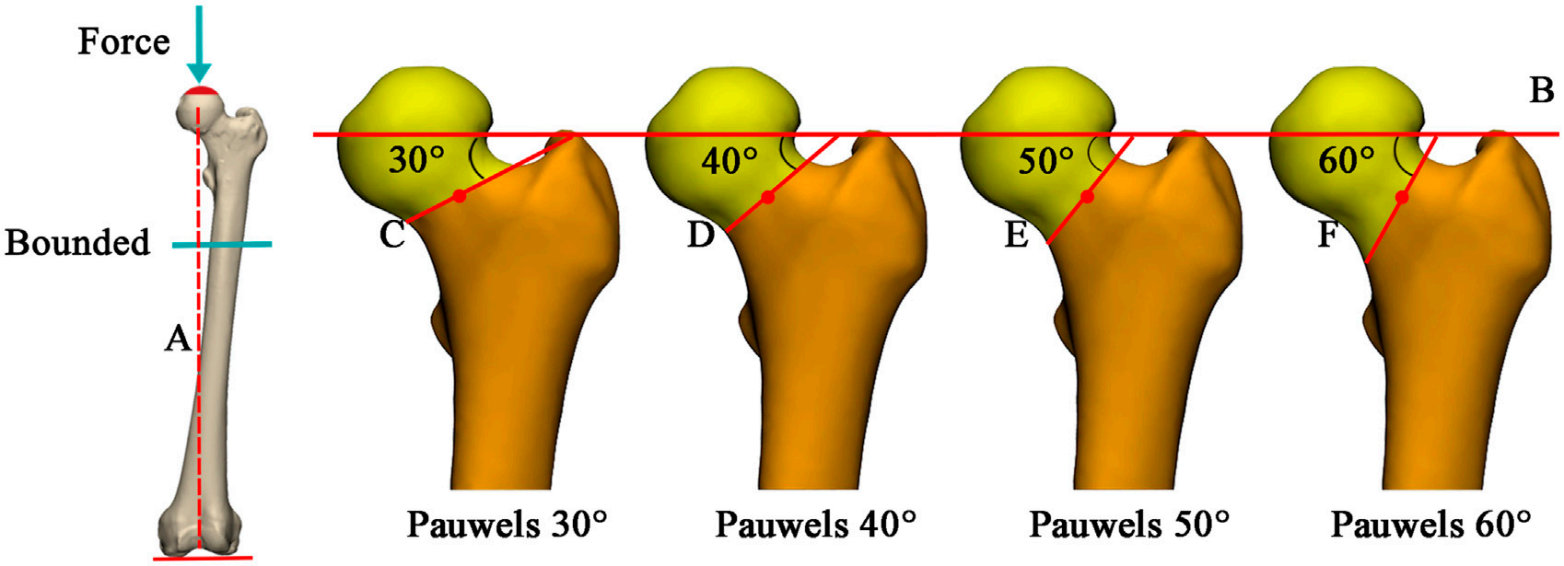
Illustration of the loading and femoral neck fracture model. **(A)** mechanical axis of the femur; **(B)** parallel lines of horizontal lines connecting the iliac crest on both sides and passing through the center of the femoral head; **(C–F)**: fracture lines of Pauwels 30°, 40°, 50°, and 60°, respectively, passing through the center of the femoral neck.

The Pauwels classification, which is vital for the biomechanical assessment of fracture healing, is based on the angle between the fracture line (C, D, E, F) and horizontal line B parallel to the iliac crest line on both sides in Figure 2. Specifically, it can be divided into three types: Pauwels Ⅰ type (< 30° ), Ⅱ type ( 30°∼50° ), Ⅲ type (> 50° ) (Bartoníček, 2001). As the number of implants increases, more blood supply in the femoral head will be destroyed, the bone loss, the operation time and cost will increase. Since Pauwels typeⅠ and Ⅱ fractures have better biomechanical stability than Pauwels type Ⅲ fractures, it is theoretically possible to maintain fixation by reducing the number of internal fixations. The scholars (Maurer et al., 2003; Krastman et al., 2006) found that patients with fine fracture reduction who were fixed with two cannulated screws had decent fracture healing. Besides, PTS have been modified and fixed based on the above biomechanical studies, and excellent clinical result has been achieved (Figure 1C) (Zhao et al., 2022).

As for Pauwels type III fractures, also known as vertical femoral neck fractures (vFNFs), due to the high vertical shear force and the fact that the tension provided by the internal fixator along the axis of the femoral neck has a component parallel to the fracture rim, an increase in the shear force at the fracture surface was observed. Thus the tension generated by the screw increased the vertical shear force on the fracture surface when CCS were used to fix the fracture, resulting in a high complication and fixation failure rate (Liporace et al., 2008). To tackle this problem, the scholars (Mir and Collinge, 2015) conceptualized incorporating a MBP into the treatment of displaced FNFs (Figure 1D). The MBP not only improved stability, but also converted the vertical shear force at the fracture surface into a compressive stress that promoted fracture healing and improved cure rates.

The healing time of FNFs and the incidence of osteonecrosis are closely related to the integrity of the internal vascular preservation of the femoral head. Therefore, ensuring tough internal fixation and protecting the remaining blood supply of the femoral head are important to avoid postoperative complications (Zhao et al., 2022). In addition, the biomechanical stability of Pauwels Ⅰ and Ⅱ fractures is better, and the stability of Pauwels Ⅲ fractures can also be improved to a certain extent after the fixation of the MBP. Therefore, we proposed that the number of internal fixations can be reduced to decrease the disruption of blood supply and the occurrence of complications, and ensure the tough fixation of the fracture. In this study, FNFs models for Pauwels 30° , 40° , 50° and 60° were created respectively. For Pauwels≤ 50° , 1, 2 and 3CCS and PTS were used to fix the fracture for the models. For Pauwels 60° , 3CCS and MBP combined with 1, 2 and 3CCS were used to fix the fracture. FE analysis was performed to verify the fixation effect and analyze the biomechanical properties, in order to provide a new therapeutic concept for clinically accurate treatment of femoral neck fractures.

## 2 Materials and methods

### 2.1 Establishment of three-dimensional femur model

A 27-year-old healthy male volunteer (175cm, 70 kg) with no history of hip or systemic disease was recruited. A Siemens 64-row spiral CT scanner was used to scan the entire femur with a thickness of 0.5 mm. The CT image was stored in the standard format of Digital Imaging and Communications in Medicine (DICOM) in Mimics 21 (The Materialise Group, Leuven, Belgium), a medical 3D reconstruction software. Appropriate gray values were selected to distinguish bone and tissue, and the three-dimensional model of the original femur was established. Then the reconstructed model was imported into 3-Matic (The Materialise Group, Leuven, Belgium) software for surface optimization processing, such as model surface defect repair, smoothing and accurate surface function.

### 2.2 Establishment of femur neck fractures model

The fracture model was established in 3-Matic software. Mechanical axis A of the femur (the line between the center point of the femoral head and the midpoint of the medial and lateral condyles of the femur on the coronal plane) was first made, then the horizontal line B, iliac crest on both sides parallel line, was made passing through the head center. The straight line C (Pauwels 30° ), D (Pauwels 40° ), E (Pauwels 50° ), F (Pauwels 60° ) intersecting B and passing through the center of the femur neck (red point in Figure 2) was drawn on the coronal plane. The fracture plane was determined by osteotomy along lines C, D, E and F perpendicular to the coronal plane. Additionally, the fracture plane was in close contact with each other with no relative displacement. In order to reduce the amount of computer calculation and save time, the middle and upper femur segments were intercepted as the test model (Figure 2).

### 2.3 Establishment of the internal fixation model

**FIGURE 3:**
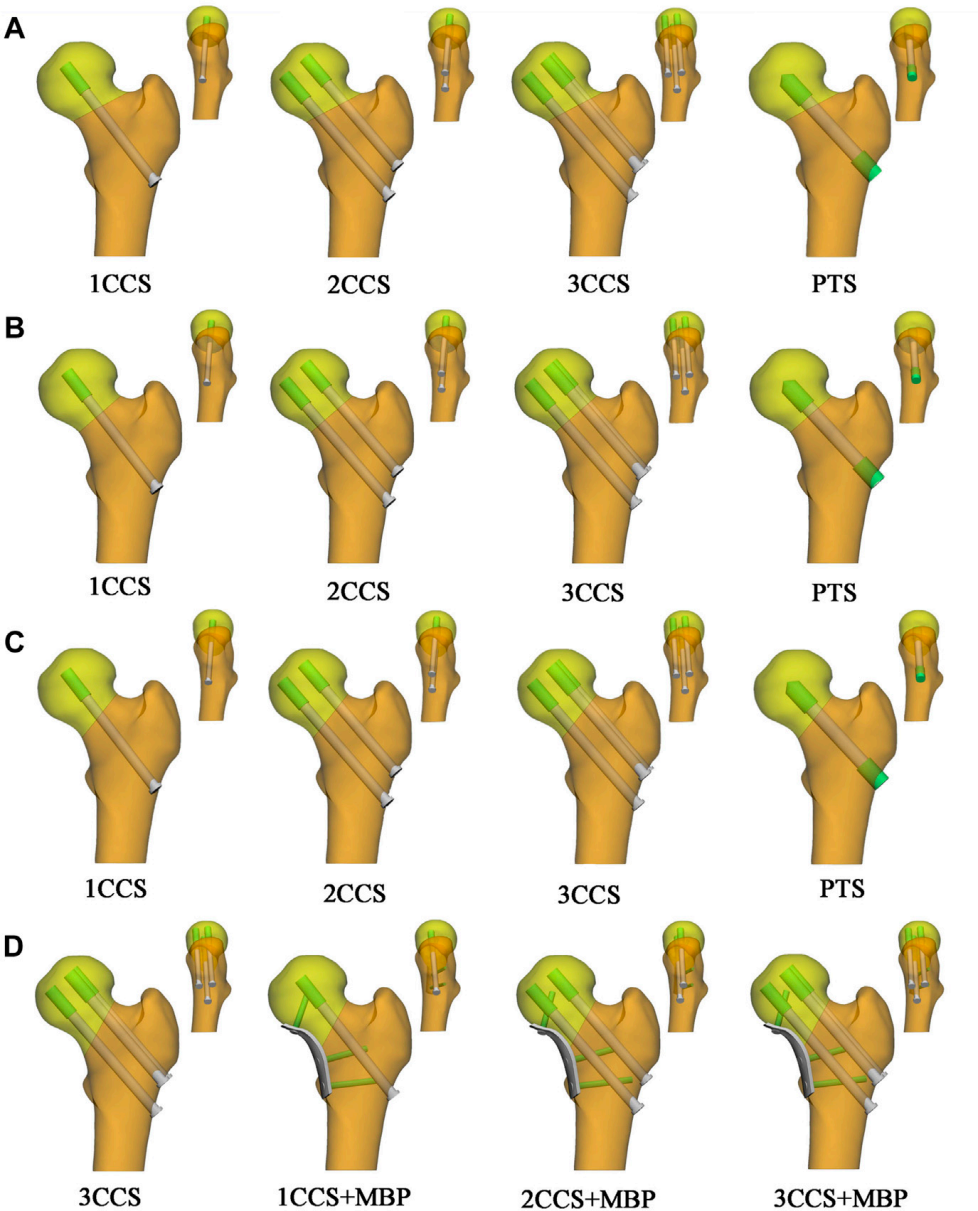
Illustration of the Non-manifold assembly FE model. **(A)** Pauwels 30°; **(B)** Pauwels 40°; **(C)** Pauwels 50°; **(D)** Pauwels 60°.

In this study, 16 fixation methods were used for 4 FNFs models. As the main purpose of this study is to investigate and compare fractures of different Pauwels classifications to select the best internal fixation mode. Experimental tests on cadaveric and on synthetic bones could provide useful information, and using full-field experimental techniques such as digital image correlation strain measurement technique (Dickinson et al., 2011) or differential thermography (Zanetti and Audenino, 2012) is highly recommended. The FE analysis method was used in this study, and it is worth noting that the focus of this study is unrelated to the thread. Therefore, to establish a FE model and facilitate calculation, all the thread portions were simplified into smooth and thick solid cylinders. The length of the thread portion was 18.0 mm, the diameter was 7.5 mm, and the diameter of the screw was 6.5 mm. The appropriate length of internal fixation was selected according to the fracture model. Firstly, in 1CCS, the screw was placed in the center of the femoral neck, and the length was 88.0 mm. Secondly, in 2CCS, the screws were fixed vertically up and down, where in the antero-posterior view, the two screws were close to the superior and inferior cortex, and where in the lateral view, the two screws were on the midline of the femoral neck. The length of the upper and lower screws were 81.0 and 90.0 mm respectively. Thirdly, in 3CCS, the screws were arranged in an inverted triangle. According to the maximum width of the three screws, the upper two screws were close to the cortex, the lower one was close to the femoral calcar. The three screws were 2.5 mm from the cortical bone and 5.0 mm from the distal subchondral bone of the femoral head, and the length of the three screws were 81, 81 and 90 mm. Fourthly, the PTS specifications were designed according to previous study (Zhao et al., 2022), similarly, the head and tail threads were simplified to a smooth and thick solid cylinder. Fifth, the thinner steel plate may have less stimulation to the internal structure of the femoral neck, so the four-hole to six-hole buttress plate is commonly used in clinic. Therefore, a MBP of 2.7 mm thickness was created in 3-Matic to fit the bone surface with reference to Stryker (Mahwah, NJ, United States) four-hole locking plate. The diameter of the plate locking screw was 3.5 mm, and the lengths were 25.0, 30.0, and 35.0 mm. The above model was Non-Fluid assembled in 3-Matic, and the effect diagram after assembly is shown in Figure 3.

### 2.4 Establishment of the FE model

FE model meshed with tetrahedral 4-nodes elements (C3D4). The average size of the proximal femur mesh was controlled to 2.0 mm, and the minimum size of the mesh was controlled to more than 1.0 mm at the bone-screw contact surface and the fracture surface of the femoral neck. To obtain both the actual structure and the calculated scale of the models, the average size of the mesh was 1.0 mm, and the average size of the body mesh of the medial buttress plate was 1.5 mm. All models being analyzed were assumed to be continuous, isotropic, and with homogeneous linear elastic materials (Sitthiseripratip et al., 2003; Taheri et al., 2011). The models were re-imported into Mimics 21 and assigned material properties according to the corresponding regions of cortical bone and cancellous bone obtained by CT scanning. This study mainly evaluated various internal fixation methods from the perspective of structure. Titanium alloy (Ti-6Al-4V) was used in all internal fixation devices to eliminate the bias caused by different materials. The parameters of various materials are listed in Table 1 (Fu et al., 2022).

**TABLE 1:** Material properties defined in the FE models.

Item	Young’s modulus (*E*, MPa)	Poisson’s ratio (*v*)	Yield stress (MPa)
Cortical bone	19,650	0.3	136.73
Cancellous bone	1,260	0.2	3.43
Ti-6Al-4V titanium	117,000	0.3	1,086
Porous tantalum	4,800	0.3	-

### 2.5 Setting of the model parameters

The above assembled models were imported into Abaqus 2021 (Simulia Corp, Providence, RI, United States), and the frictional contact interactions were set according to previous studies: the screws, MBP and bone was 0.3, and the interaction between fracture surface was 0.46 (Eberle et al., 2010). Besides, the binding relationship between thread, plate locking screw and bone was set. No pre-strain given by the screws between the two bone fragments was set. All nodes on the distal femur surface were constrained with 0 degrees of freedom to prevent rigid body movement during analysis. From the previous studies, when walking or running on the ground, the combined force exerted by human gravity and the muscles and ligaments around the hip joint can reach 3 to 5 times of the body weight (Cha et al., 2019). Therefore, in the present analysis, a load of 2100N corresponding to 3 times the body weight was uniformly applied to the main weight-bearing area of the femoral head along the mechanical axis of the femur as shown in Figure 2.

### 2.6 Evaluation criteria

Firstly, the evaluation parameters of MBS, MIS, MBD were measured. Secondly, since stiffness may not accurately reflect the stability around the fracture site, we did not use it as an evaluation parameter in the analysis of experimental results. Instead, interfragmentary motion (IFM) at the fracture end was used as an evaluation parameter. It can reflect the real “stability” of the model, which was the ultimate embodiment of stiffness and directly affects the healing effect. Previous studies have suggested that the optimal micromovement amplitude to accelerate fracture healing was within 0.5 mm (Kenwright and Goodship, 1989; Wolf et al., 1998).

## 3 Result

### 3.1 Validation of the developed FE model

Pauwels 60° 3CCS and 3CCS + MBP models were compared with previously published data to evaluate the validation of the FE models. The results from Li et al. (Li et al., 2019a) (MBS:116.32 MPa; 114.91 MPa) and Zhan et al. (Zhan et al., 2020) (MBS: 138.8 MPa; 118.4 MPa) were compared with our study (MBS: 128.6 MPa; 113.9 MPa). The results are similar, which verifies that the FE model in our study is suitable for further analysis.

### 3.2 The von mises stress distribution

**FIGURE 4:**
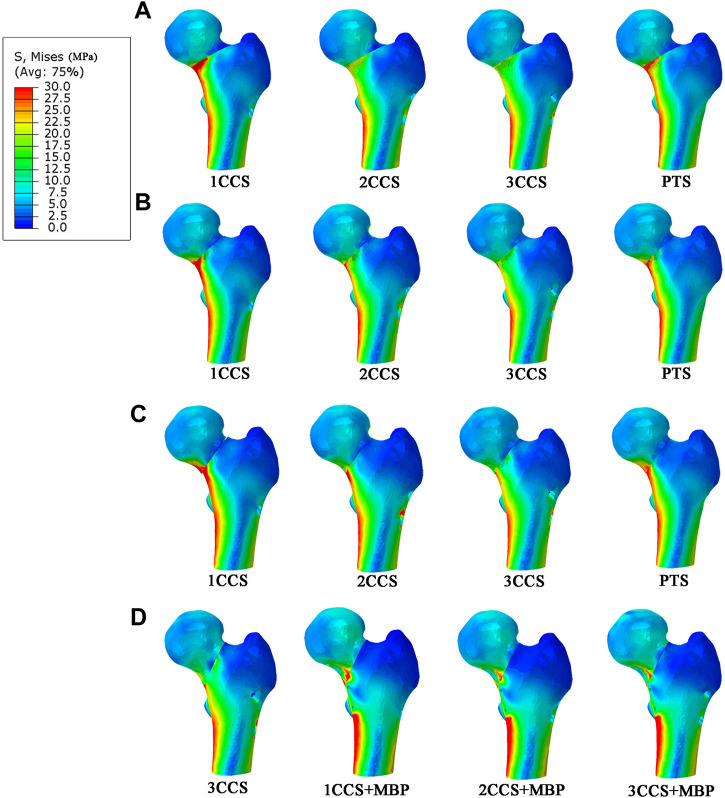
Stress distribution in different femur models.**(A)** Pauwels 30°; **(B)** Pauwels 40°; **(C)** Pauwels 50°; **(D)** Pauwels 60°.

**FIGURE 5:**
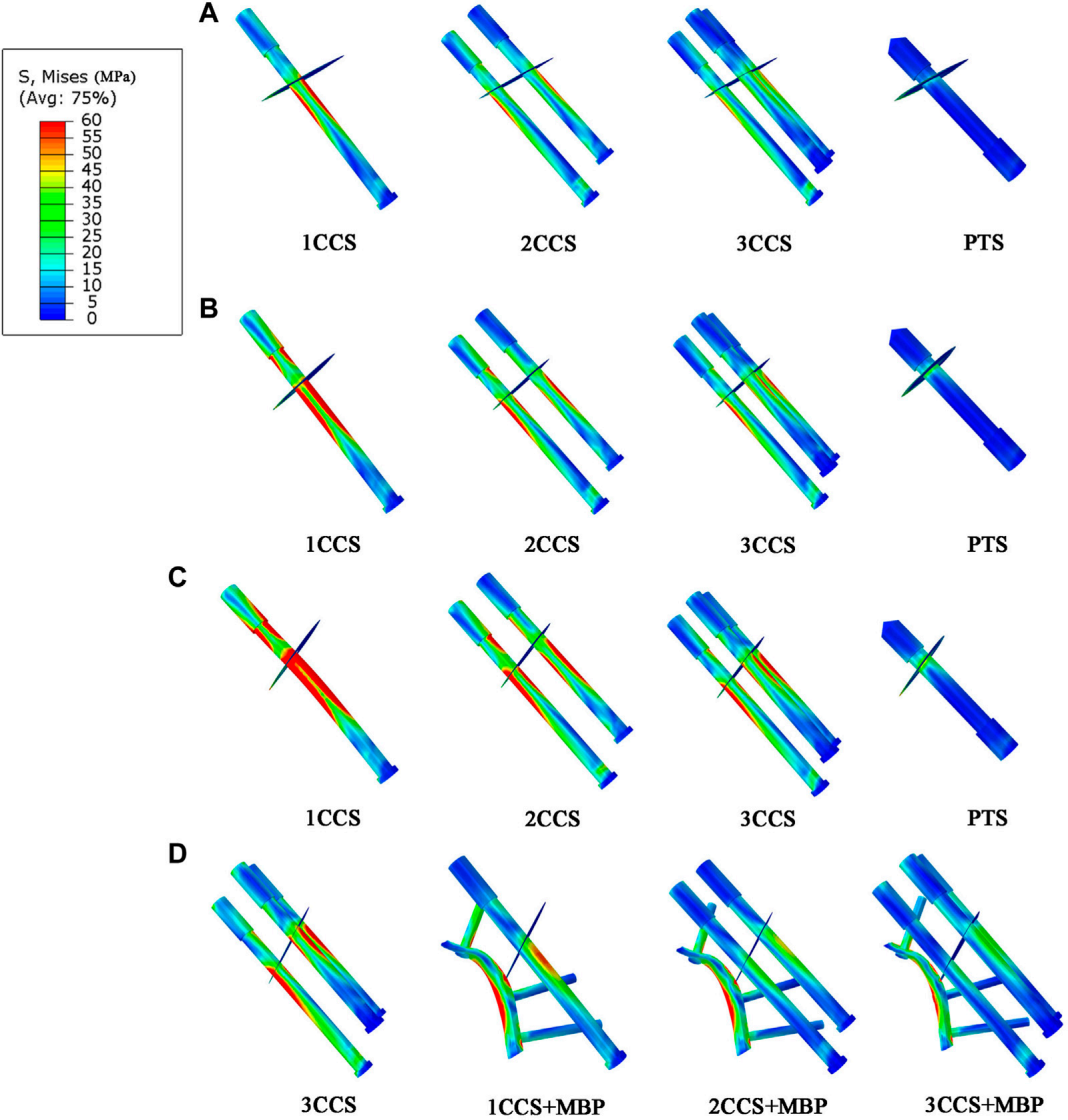
Stress distribution in different internal fixation models. **(A)** Pauwels 30°; **(B)** Pauwels 40°; **(C)** Pauwels 50°; **(D)** Pauwels 60°.

In the models with different Pauwels angles, it was observed that the femoral stress was mainly concentrated at the fracture end and the medial cortex of the femur (Figure 4), and the internal fixation stress was mainly concentrated at the screw near the fracture surface and the junction between the plate and the fixation screws (Figure 5). In the same angle fracture, the stress became more distributed and balanced with the increase of the number of internal fixations. In the same internal fixation group, with the increase of Pauwels angle, the maximum stress on the femur and the internal fixation also increased.

At Pauwels 30° , the maximum stress was found in 1CCS, which was 94.8 MPa in the femur and 307.7 MPa in internal fixation. In the Pauwels 40° and 50° groups, the stress on the medial cortex of the femoral fracture and the screws gradually increased. At Pauwels 50° , the maximum stress of 1CCS was 1,195.3 MPa (exceeding the yield strength of the screw), and the others were less than the yield range of the material. In the PTS group, the maximum femur stress was between 1CCS and 2CCS, and the maximum internal fixation stress was less than that in the 2CCS group.

At Pauwels 60° , the femur stress of 3CCS group was 128.6 MPa (close to the yield strength of the femur cortex), and the internal fixation stress was mainly concentrated under the femoral calcar screw, with an average of 326.1 MPa. After adding the MBP, the maximum stress of the femur was mainly concentrated in the contact area between the fracture end and the plate. Since the steel plate bore more of the stress, the CCS stress was significantly reduced. The MBS of 1CCS + MBP, 2CCS + MBP, 3CCS + MBP groups were 138.93 (exceeding the yield strength of cortical bone), 124.2, and 113.9 MPa, respectively (Table 2).

**TABLE 2:** Parameters results of FE models.

Pauwels	IF	MBS (MPa)	MIS (MPa)	MBD (mm)	IFM (mm)
30°	1CCS	94.8	307.7	0.86	0.36
	2CCS	86.1	254.4	0.73	0.27
	3CCS	65.2	174.3	0.69	0.15
	PTS	81.6	126.8	0.71	0.31
40°	1CCS	108.5	679.6	0.98	0.58
	2CCS	90.2	507.9	0.76	0.43
	3CCS	70.6	282.4	0.72	0.27
	PTS	92.3	264.3	0.75	0.36
50°	1CCS	130.5	1,195.3	1.29	1.12
	2CCS	116.2	741.1	0.83	0.65
	3CCS	102.7	308.5	0.77	0.34
	PTS	118.5	345.6	0.81	0.45
60°	3CCS	128.6	326.1	0.83	0.63
	1CCS + MBP	138.9	772.6	0.78	0.66
	2CCS + MBP	124.2	602.5	0.75	0.48
	3CCS + MBP	113.9	319.8	0.72	0.16

Abbreviations: IF: internal fixation; MBS: maximal stress of the bone; MIS: maximal stress of the implants; MBD: maximal displacement of bone; IFM: interfragmentary motion.

### 3.3 The displacement distribution of different femur models

**FIGURE 6:**
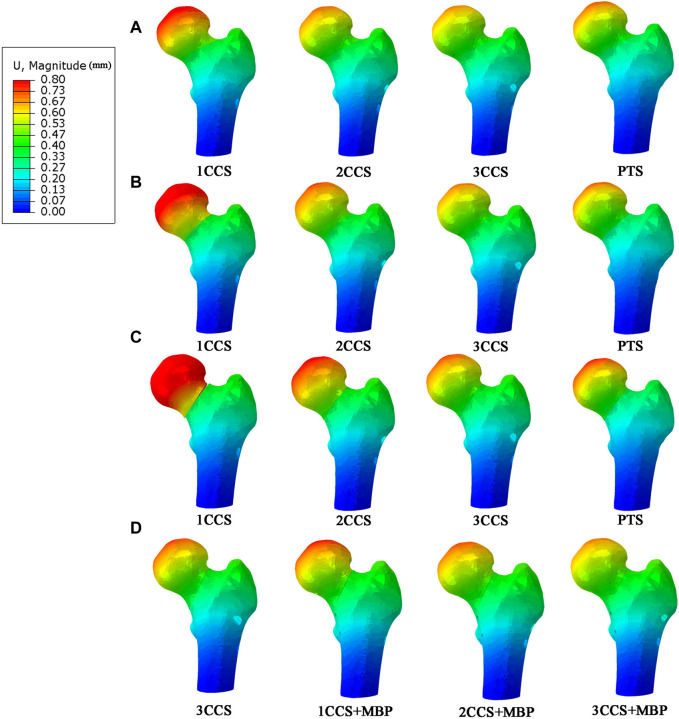
Displacement distribution in different femur models. **(A)** Pauwels 30°; **(B)** Pauwels 40°; **(C)** Pauwels 50°; **(D)** Pauwels 60°.

The displacement distribution of the femur was shown in Figure 6. It can be observed from the figure that the displacement gradually increased from the distal end of the femoral model to the femoral head, and the largest displacements were located at the femoral head where the load was applied. It is worth noting that a discontinuity in displacement pattern is a clear sign of the fracture undergoing tensile stresses. Besides, the femoral displacement of the 1CCS group was the largest, and the maximum displacement decreased with the increase in the number of CCS. When using the same internal fixation, the MBD increased with the increase of the Pauwels angle. In the PTS group, the MBD was between 2CCS and 3CCS, which was close to the 2CCS fixation effect.

At Pauwels 60° , the MBD in the three groups with MBP was significantly reduced compared with the 3CCS fixation group, indicating that the addition of the MBP could obviously enhance the stability of the models.

### 3.4 Interfragmentary motion of femur neck fracture

**FIGURE 7:**
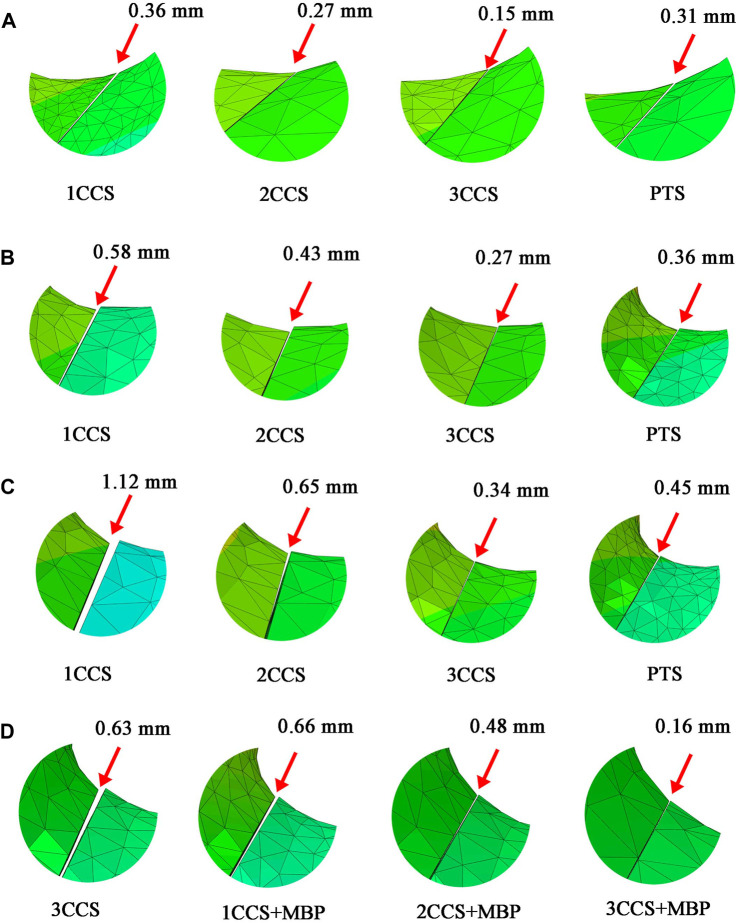
Interfragmentary motion of femur neck fracture. **(A)** Pauwels 30°; **(B)** Pauwels 40°; **(C)** Pauwels 50°; **(D)** Pauwels 60°.

The interfragmentary motion diagram was shown in Figure 7. At Pauwels 30°∼50° , the 1CCS group had the largest relative displacement of the fracture fragment. The results showed that the IFM increased with the increase of Pauwels angle when the same internal fixation was used. In the same fracture type, with the increase in the number of fixed CCS, the IFM decreased. Besides, the fixation effect of the PTS group was like that of the 2CCS group, indicating the excellent fixation advantage of the PTS.

At Pauwels 60° , the IFM of the 3CCS group was 0.63 mm. However, the IFM in the model with MBP was significantly reduced. The results showed that the MBP was crucial to improve the overall stability of unstable fractures.

## 4 Discussion

FNFs in non-elderly patients are usually treated with hip preservation, but the treatment method is still controversial. With an improved understanding of fracture biomechanics, clinicians have been able to better understand and prevent these complications. Especially, FE analysis was the most widely used method for stress analysis in biomechanical research, which can provide a reliable basis for the selection of internal fixation methods for FNFs.

In recent years, the innovation of internal fixation methods for FNFs has become a research hotspot. There are many innovative and improved methods of internal fixation for Pauwels type Ⅲ fractures. However, the excessive pursuit of the stability of internal fixation leads to the aggravation of the damage to the blood supply of the femoral head. In fact, the biomechanics of fractures with different Pauwels angles are completely different. In this study, we selected appropriate internal fixation methods based on different Pauwels angles and verified their fixation effects. Thus, the difference of the different models could be investigated and the results could provide guidance for the selection of clinical implantation scheme.

From the results of this study, it can be observed that the fracture of Pauwels type Ⅰ (< 30° ) had relatively more contact with the fracture surface and biomechanical stability. When using 1CCS fixation, MBS and MIS did not exceed the yield strength of the material, and the IFM was 0.36 mm, indicating that this method of fixation could fulfill the biomechanical requirements of fracture healing. From the results of this study, it can be illustrated that the stress and displacements of the model fixed by PTS were like those fixed by 2CCS group. Pauwels type I fracture line was relatively horizontal, the horizontal component (shear force) of the resultant force acting on the fracture line was relatively small, and the friction force on the fracture surface could counteract or weaken the shear force, so in fact the fracture was a stable fracture. The results of this study indicated that for Pauwels type Ⅰ fracture, reducing the number of CCS can also maintain the mechanical environment that can promote fracture healing. PTS were designed based on the above results, researchers optimized CCS to increase the diameter of the screws, and reduce the length and number of screws to decrease the damage to the blood supply of the femoral head. Besides, compared with traditional porous metals, porous tantalum had structural properties similar to the subchondral bone, with a higher friction coefficient and higher initial stability. And it had higher porosity, which promoted bone growth and achieved long-term stability. PTS not only reduced the number of internal fixation placement and promoted fracture healing, but also effectively protected the fragile blood supply in the femoral head, so as to reduce the incidence of postoperative complications. In particular, the PTS fixation method was an appropriate alternative and had proven clinical effectiveness (Zhao et al., 2022).

For the fracture of Pauwels type Ⅱ ( 30°∼50° ), the internal fixation stress was more focused when 1CCS was used for fixation. As the Pauwels angle increased, the vertical shear force at the fracture surface also increased, the MBS became more concentrated in the cortex below the fracture, and the MIS also became more concentrated, where refracture and internal fixation crack may occur. However, in the 2CCS, 3CCS, PTS groups, where all the evaluation parameters were reduced, the fixation effect of PTS was like that of 2CCS group, and the stress, displacement and IFM could fulfill the requirements of fracture healing. Although the results of this study suggest that the 2CCS and PTS groups had a satisfactory fixation effect, the binding relationship between screw threads and bone may lead to osteoporosis or other clinical problems. Further clinical verification is needed for the fixation effect of 2CCS and PTS. To be on the safe side, we still recommend using 3CCS for fixation based on accurate reduction. Since the clinical problems still need to be solved, new internal fixation methods that could improve fixate stability and reduce blood supply destruction need to be further studied.

For Pauwels type Ⅲ fractures (> 50° ), vertical shear force was dominant, and internal fixation must be able to resist it. However, none of the existing fixation methods can provide an anti-shear force buttress at the medial side of the femoral neck. Although using 3CCS had less trauma, small incision, and less intraoperative blood loss, some previous studies have reported that this method has insufficient biomechanical stability and more postoperative complications (Ma et al., 2018). The incidence of fracture non-union has been reported to be 19% (Parker, 2009). To solve this problem, DHS and other internal fixation methods have been developed. However, biomechanical experiments indicated that those internal fixation methods had insufficient anti-shear effects (Aminian et al., 2007). But the MBP was placed on the medial side of the proximal femur, which not only had anti-sliding and anti-rotation properties, but also can transform the shear force of the fracture into the compressive stress that promoted fracture healing, providing a satisfactory biomechanical environment for fracture (Tianye et al., 2019). In addition, incision of the joint capsule during MBP placement can reduce intra-capsular hematoma, promote blood circulation in the femoral head, and reduce the occurrence of postoperative osteonecrosis (Mir and Collinge, 2015). However, some scholars (Lazaro et al., 2013) believed that the inferior retinacular artery (IRA) may be injured during the process of MBP implantation, which plays an important role in femoral head perfusion. In fact, the IRA was in the posterior interior of the femoral neck. If the MBP was placed in the anterior medial side of the femoral neck through the modified anterior approach, the IRA might not be injured, and the fracture could be anatomically reduced, which could reduce postoperative complications.

In this study, the MBS in the 3CCS group was close to its yield strength. Besides, the stress was concentrated in the contact part of the femur and the lower screw tail, which had the risk of nail withdrawal. The IFM of this group was 0.63 mm, indicating that the fracture fragments were relatively displaced too much, and the bone was prone to non-union in the long term. However, CCS was combined with MBP, which distributed stress on CCS by providing an additional route to transfer stress between fracture fragments. In the 2CCS + MBP group, the IFM was less than 0.5mm, and the other parameters were significantly better than those of the 3CCS group. Furthermore, it can be observed in Figure 4 that the maximum stress was concentrated below the fracture surface of the femoral neck in contact with the plate. The reason for this phenomenon was that the fracture of the neck changed the mechanical conduction in the proximal femur, and the load in the proximal femur was mainly transmitted by these screws and plates. After adding the MBP, the stress of CCS and the medial femoral neck region was significantly reduced, indicating that the plate played a crucial role in supporting the fracture and establishing a better biomechanical environment for fracture healing.

Although we have comprehensively evaluated the different implantation approaches, there are still some limitations in this study. First, all models were developed using linear elastic materials, and the relationship between thread and bone was set as a binding relationship, without bone plastic deformation or screw loosening process. This study only focused on the initial stability, rather than the stability during bone healing. On the other hand, considering that the initial stability is crucial (Aminian et al., 2007) for fracture healing, the results of this study are still meaningful. Second, the intact fracture surface and theoretical anatomical reduction in our model, as well as a null pre-strain given by the screws between the two bone fragments, which may affect the accuracy of the results. Third, the thread of the implant was simplified in this study, but it proved to have little effect on the results (Inzana et al., 2016). Fourth, we only applied forces along the mechanical axis of the femur and did not simulate the muscles attached to the femur (such as action of abduction muscles), which may not accurately reflect the proximal femoral movement in the physiological loading mode and may affect the internal fixation stress distribution. In general, further biomechanical experiments research in cadavers is still necessary in the future. Despite these limitations, our results may help orthopedic surgeons to select the most appropriate fixation strategy in clinical practice.

## 5 Conclusion

When the Pauwels angle is small, the fracture position has a compressive effect, which is conducive to fracture healing. Therefore, Pauwels type Ⅰ (< 30° ) fractures can reduce the number of internal fixations, and PTS is a better alternative treatment option. For Pauwels type Ⅱ fractures ( 30°∼50° ), the 3CCS fixation method is still recommended. However, new internal fixation methods that can improve fixation stability and reduce blood supply destruction need to be further studied in the future. As the Pauwels angle increases, greater shear forces lead to an increased risk of fracture displacement and non-union. For Pauwels type Ⅲ fractures (> 50° ), it is recommended to add MBP to the medial femoral neck and combine it with 2 CCS to ensure a stable fracture healing environment is established.

## Data Availability

The original contributions presented in the study are included in the article/Supplementary Material, further inquiries can be directed to the corresponding authors.
